# The first complete mitochondrional genome of *Anopheles gibbinsi* using a skimming sequencing approach.

**DOI:** 10.12688/f1000research.148473.1

**Published:** 2024-05-30

**Authors:** Renee Ali, Mary E. Gebhardt, James Sichivula Lupiya, Mbanga Muleba, Douglas E. Norris

**Affiliations:** 1 The W. Harry Feinstone Department of Molecular Microbiology and Immunology, Johns Hopkins Malaria Research Institute, Johns Hopkins Bloomberg School of Public Health, Baltimore, Maryland, USA; 2 Tropical Diseases Research Centre, Ndola, Zambia

**Keywords:** Anopheles gibbinsi, understudied anopheline, genome skimming, Zambia

## Abstract

Mosquitoes belonging to the genus *Anopheles* are the only vectors of human malaria. *Anopheles gibbinsi* has been linked to malaria transmission in Kenya, with recent collections in Zambia reporting the mosquito species exhibiting zoophilic and exophilic behavioral patterns with occasional contact with humans. Given the paucity of genetic data, and challenges to identification and molecular taxonomy of the mosquitoes belonging to the *Anopheles* genus; we report the first complete mitochondrial genome of *An. gibbinsi* using a genome skimming approach. An Illumina Novaseq 6000 platform was used for sequencing, the length of the mitochondrial genome was 15401 bp, with 78.5% AT content comprised of 37 genes. Phylogenetic analysis by maximum likelihood using concatenation of the 13 protein coding genes demonstrated that *An. marshallii* was the closest relative based on existing sequence data. This study demonstrates that the skimming approach is an inexpensive and efficient approach for mosquito species identification and concurrent taxonomic rectification, which may be a useful alternative for generating reference sequence data for evolutionary studies among the Culicidae.

## Introduction

A few species in the genus *Anopheles* are well established primary vectors of human malaria in sub-Saharan Africa, including *Anopheles gambiae* and *An. funestus.* However, studies have also implicated understudied anopheline mosquito species in driving transmission in regions where primary vectors are close to elimination. ^1^^–^^3^ *Anopheles gibbinsi*, until recently recognized as *An. species 6*, has tested positive for *Plasmodium falciparum* sporozoites in studies from Kenya. ^4^^,^^5^ Furthermore, *An. gibbinsi* was previously reported from central, eastern and northern Africa and has now been shown to have a geographic range extending into southern Africa. Recent first-time captures for this species in Zambia reported the species largely exhibiting zoophilic and exophilic behavioral patterns; however, a blood meal PCR assay also detected a few specimens positive for human host DNA. ^5^ Similar in morphology to other well-established malaria vectors and with a dearth of genetic data available, there is a need for the continued monitoring of *An. gibbinsi* as a potential vector in malaria transmission. It is also important that tools for accurate mosquito identification be developed, due to limitations with the commonly targeted cytochrome oxidase I gene (COI) and the internal transcribed spacer 2 (ITS2) in resolving members of morphologically cryptic species complexes. ^6^

The expansion of sequencing strategies has employed the use of mitochondrial genomes (mitogenomes) primarily for species identification and solving discrepancies in the taxonomic classification of metazoan organisms. Mitogenomes have also proved useful in evaluating population structure, chromosomal rearrangements, species introgression, and evolutionary histories. ^7^^–^^9^ The mitochondrial DNA (mtDNA) is a circular double stranded molecule that encodes 37 genes made up of 13 protein coding genes (PCGs), 22 transfer RNA (tRNA) genes), 2 ribosomal RNA (rRNA) genes and an adenine and thymine (A-T) rich terminal, an essentially non-coding area termed the control region (CR) associated with the replication and transcription of the genome. Maternal inheritance, high copy number, lack of recombination, and absence of introns are characteristics which allow mtDNA to be well suited for accurate molecular identification and rectifying taxonomic classification among species. ^6^^,^^10^ Here for the first time, we describe a genome skimming approach for recovery of, and characterization of the mitochondrial genome of *An. gibbinsi* and its phylogenetic relationship to other established anopheline vectors of human malaria.

## Methods

### DNA collection

The *An. gibbinsi* specimens (n = 3) sequenced were collected in Nchelenge, Zambia using a CDC light trap that was placed near an animal. The specimens were stored on silica gel until DNA extraction. Single mosquito specimens were pre-treated ^11^ as described by Chen *et al*. 2021, followed by the extraction protocol as per manufacturer’s instructions (Qiagen DNeasy Blood and Tissue Kit, Hilden, Germany). A whole mosquito specimen was placed in a 1.5 mL Eppendorf tube and homogenized in a cocktail containing 98 μL of PK buffer (Applied Biosystems, Waltham, Massachusetts, U.S.A.) and 2 μL of Proteinase K (100 mg/mL), this was followed by an incubation step for 3 hours at 56 ^o^C. ^12^ After incubation, 100 μL of isopropanol and 100 μL of Buffer AL (Qiagen DNeasy Blood and Tissue Kit, Hilden, Germany) was added to the lysate and left to incubate at room temperature for 10 minutes. The mixture was pipetted into a DNeasy mini spin column placed in a 2 mL collection tube; extraction protocol was followed ^5^and stored at -20 ^o^C prior to sequencing. DNA was shipped to SeqCenter (Pittsburg, U.S.A) for library construction and sequencing. Libraries were sequenced from both ends (150 bp) on Illumina Novaseq 6000 to a depth of 13.3 million reads.

**Figure 1.**
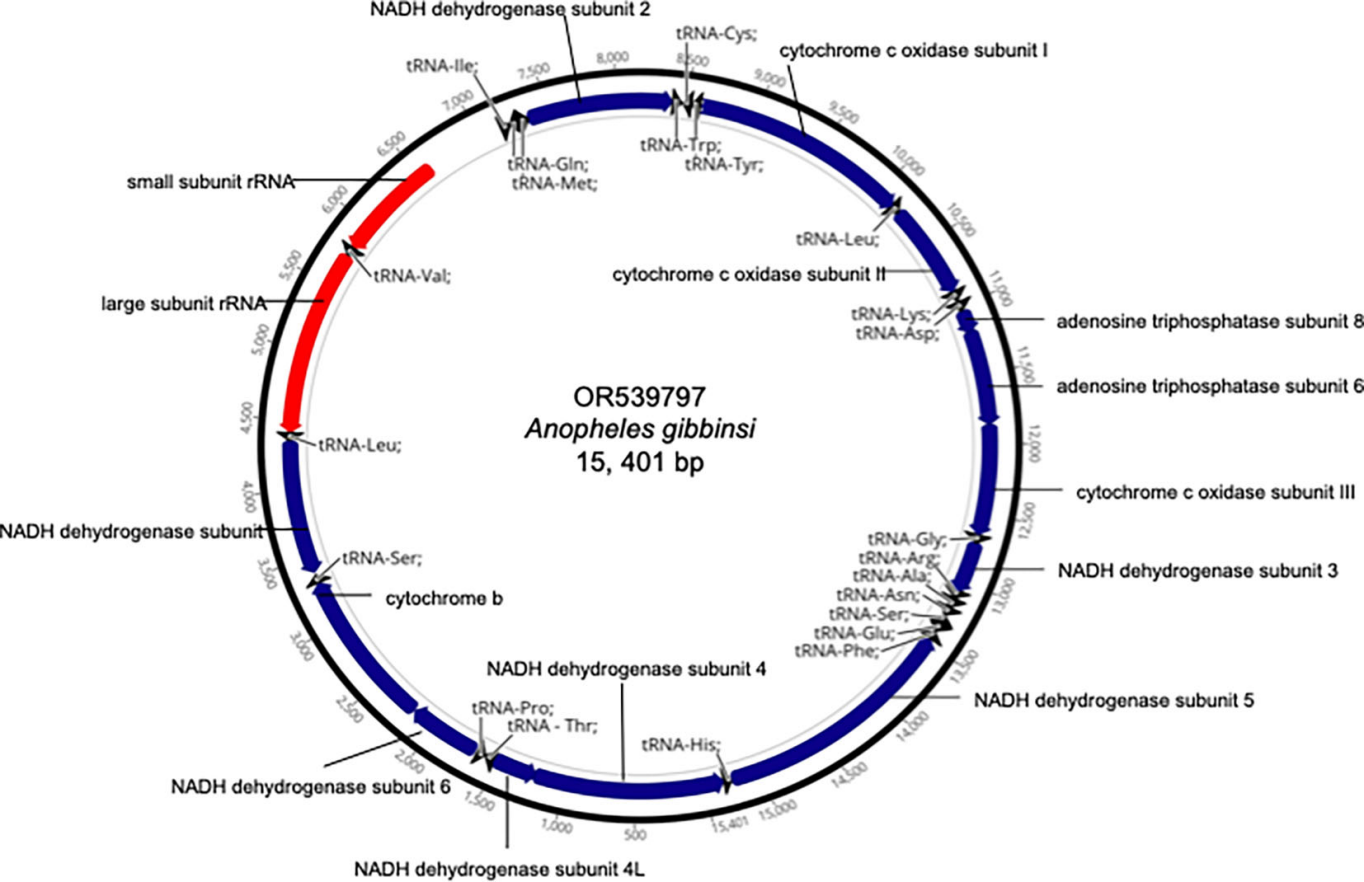
Mitogenome map of *Anopheles gibbinsi* with annotated genes.

The mitochondrial genome contigs were assembled similar to that of the *An. squamosus* ^13^ mitogenome using NOVOPlasty (RRID:SCR_017335) version 4.3.1. ^12^ Using the invertebrate genetic code under default settings, automatic annotations were conducted using the MITOS website. ^14^ Adjustments for start and stop codon positions were performed manually in Geneious Prime (RRID:SCR_010519) version 2023.2.1 (Biomatters, Auckland, Australia) to match reference anopheline mitogenomes deposited in NCBI’s GenBank database. The mitochondrial genome sequences and their corresponding annotations were submitted to the GenBank database. A representative mitogenome map is provided in Figure 1.

Phylogenetic analysis was performed using the concatenated 13 PCGs of the three *An. gibbinsi* specimens, eight *Anopheles* species and one *Aedes* species as an outgroup. The General Time Reversible (GTR + G + 1) model was identified as the best fit for building the maximum likelihood phylogenetic tree in MEGA (RRID **_**SCR_023017) version 11 ^15^ using 1000 bootstrap replicates.

## Results

The sequencing from the 3 *An. gibbinsi* samples yielded an average of 29,674,206 million reads and of these, approximately 119,364 reads were used to assemble each mitochondrial genome. The contents of the 3 *An. gibbinsi* mitogenomes (GenBank accession numbers OR_539796, OR_539797, OR_569715) included 2 ribosomal RNAs, 22 transfer RNAs and 13 protein coding genes. The representative mitochondrial genome (OR_539797) length was 15,401 bp with an A + T percentage of 78.5% which is comparable to other anopheline mitogenomes deposited in the GenBank database. The cytochrome c oxidase I (COI) fragment spanning 8598–10,133 bp was 94.5% similar to a COI sequence for *An. marshallii* (GenBank YP_010419919).

**Figure 2.**
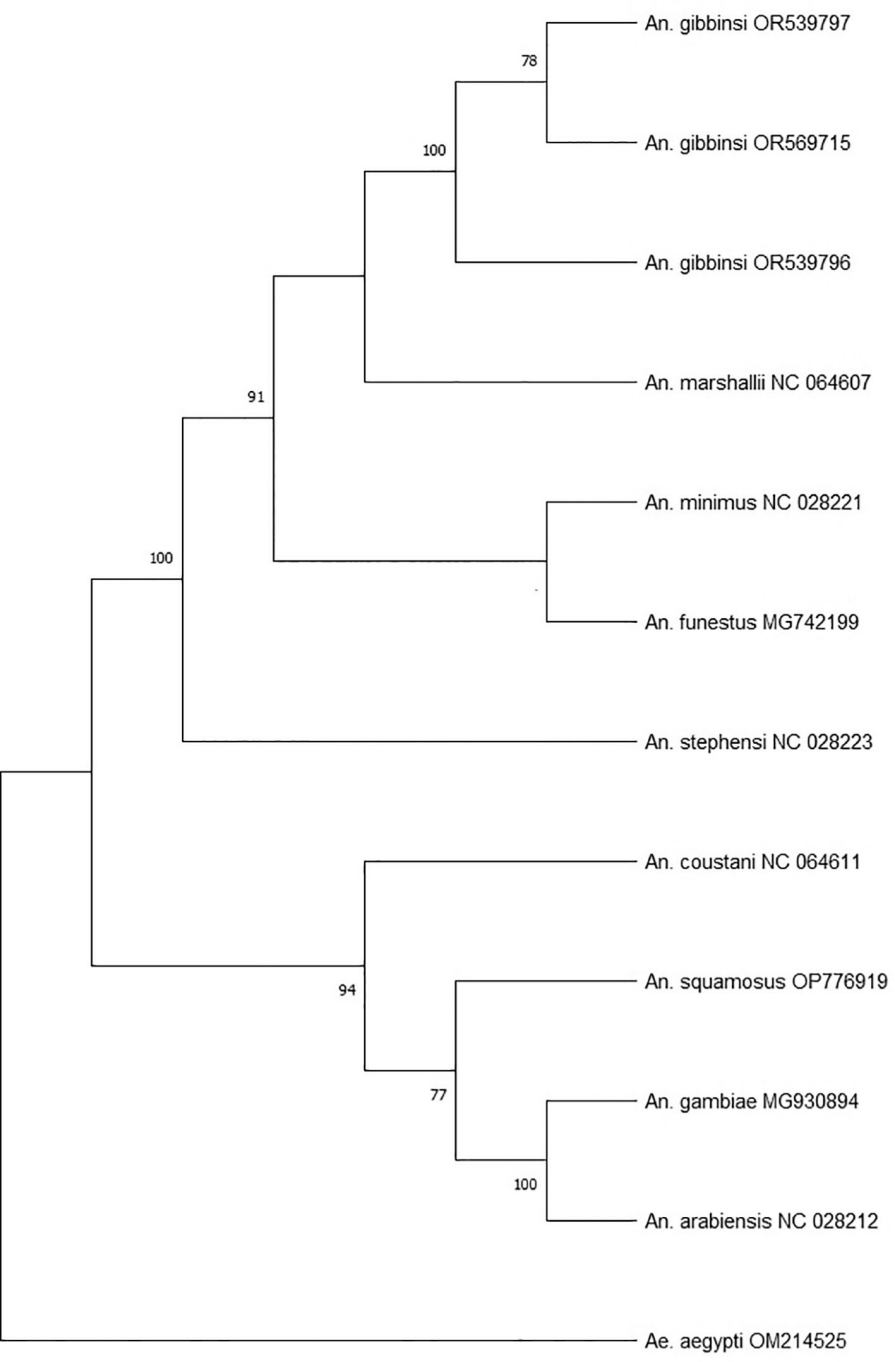
Maximum likelihood tree using the concatanated protein coding genes of *An. gibbinsi* and related *Anopheles.*

Phylogenetic analysis ( Figure 2) using the concatenated PCGs revealed *An. marshallii* (NC_064607) as the closest sequenced relative to *An. gibbinsi,* forming a single but weakly supported clade apart from the well-recognized vectors of malaria. Both species belong to the *An. marshallii* complex.

Genome skimming has demonstrated to be a cost-effective approach for generating reference sequence data which can be used for mosquito identification and resolving phylogenies. With the continued monitoring of *An. gibbinsi* as a potential vector for malaria transmission, this study provides a key genomic resource for understanding the phylogenetic relationship of this mosquito species within its complex and with primary vectors of human malaria transmission.

## Data Availability

GenBank: *Anopheles gibbinsi* mitochondrion **,** complete genome. Accession numbers OR539796, OR539797, OR569715. ^16^ Bio Project. Complete mitogenome sequence of *Anopheles gibbinsi* from Nchelenge, Zambia, Accession number PRJNA1072262. ^17^ SRA. Illumina seq of *Anopheles gibbinsi.* Accession numbers SRR27842795, SRR27842796, SRR27842794. ^18^ BioSample: *Anopheles gibbinsi* isolates ANGB, AGB1, GN. SAMN39739077, SAMN39739078, SAMN39739079. ^19^
