# Supplementary material for: The first complete mitochondrional genome of Anopheles gibbinsi using a skimming sequencing approach
Source: F1000Res. 2024 May 30;13:553. [Version 1] doi: 10.12688/f1000research.148473.1 (PMC11258543; doi:10.12688/f1000research.148473.1)
Supplement: Peer review history

# Reviewer report: Reviewer response for version 1

**DOI:** 10.5256/f1000research.162785.r298679

**Published:** 2024-07-22

**Authors:** Alexey Makunin

**Affiliations:** 1 Wellcome Sanger Institute, Hinxton, England, UK

The manuscript by Ali et al presents three mitogenome assemblies for Anopheles gibbinsi, a potential secondary malaria vector mosquito species, generated with low coverage shotgun Illumina sequencing. Overall, the data presentation and text quality are good. I only have several technical comments:

 Introduction

 "_Anopheles gibbinsi_, until recently recognized as _An. species 6_," - unclear what this means

 "The mitochondrial DNA (mtDNA) is a circular double stranded molecule that encodes 37 genes" - genome structure and number of genes varies across eukaryotes - I'd suggest specifying a taxon here

 Methods

 Did you do any morphological species identification before extraction?

 Did you do any quantification, fragment size and purity estimation for the DNA before library prep?

 There is a discrepancy in read counts reported in methods and results: "Libraries were sequenced from both ends (150 bp) on Illumina Novaseq 6000 to a depth of 13.3 million reads." vs "The sequencing from the 3 An. gibbinsi samples yielded an average of 29,674,206 million reads"

 It might be useful to provide a summary table with sequencing statistics per sample: pre-sequencing metrics (if any available, e.g. total amount of DNA used for library construction or average fragment size), total number of reads, number of reads used in the assembly, assembled mitogenome size before and after correction

 "cytochrome c oxidase I (COI) fragment spanning 8598–10,133" - in https://www.ncbi.nlm.nih.gov/nuccore/OR539797, span of COI is 8595..10128

 Fig 2 - " forming a single but weakly supported clade" - support value for marshalli-gibbinsi group is not shown on the tree

 "Both species belong to the _An. marshallii_ complex" - online databases like WRBU and Mosquito Taxonomic Inventory suggest that An. gibbinsi is in marshalli group, not complex. Might be useful to add a reference here.

Are the datasets clearly presented in a usable and accessible format, and the assembly and annotation available in an appropriate subject-specific repository?

Yes

Are sufficient details of the sequencing and extraction, software used, and materials provided to allow replication by others?

Yes

Are the rationale for sequencing the genome and the species significance clearly described?

Yes

Are the protocols appropriate and is the work technically sound?

Yes

Reviewer Expertise:

comparative genomics

I confirm that I have read this submission and believe that I have an appropriate level of expertise to confirm that it is of an acceptable scientific standard.

# Reviewer report: Reviewer response for version 1

**DOI:** 10.5256/f1000research.162785.r298674

**Published:** 2024-07-18

**Authors:** Luke Ambrose

**Affiliations:** 1 The University of Queensland, Saint Lucia, Queensland, Australia

This is a useful report of an additional resource for identifying a cryptic species of *Anopheles*. I have a few suggestions that may improve the study:

 1) Mitochondrial DNA alone is not sufficient in most cases to delineate species due to the potential for introgression. This is commonly observed in many diverse organisms. I suggest at minimum a discussion of this. Developing a supporting nuclear based diagnostic, such as an ITS2 RFLP would be preferable and should not be too expensive or difficult. Additionally, it would be useful to identify regions of the mitogenome that may differ between closely related species in the complex and to perform sequencing on samples from across Africa however that could be in a follow up study. 

 2) The language in the methods is a bit unclear - was the raw data mapped to the An. squamosus mitogenome? If so please clarify wording in line with this. I would also like to know average sequencing depth of the mapped genomes rather than reporting 13.3 million reads (this isn't actually depth I assume but how many read mapped to mitogenomes?)

Are the datasets clearly presented in a usable and accessible format, and the assembly and annotation available in an appropriate subject-specific repository?

Yes

Are sufficient details of the sequencing and extraction, software used, and materials provided to allow replication by others?

Partly

Are the rationale for sequencing the genome and the species significance clearly described?

Yes

Are the protocols appropriate and is the work technically sound?

Partly

Reviewer Expertise:

Population genetics of Anopheles mosquitoes.

I confirm that I have read this submission and believe that I have an appropriate level of expertise to confirm that it is of an acceptable scientific standard.

# Reviewer report: Reviewer response for version 1

**DOI:** 10.5256/f1000research.162785.r288704

**Published:** 2024-06-21

**Authors:** Nicola Zadra

**Affiliations:** 1 NBFC, Palermo, Italy; 2 University of Trento, Trento, Italy

The paper is well-written, and the message delivered is thorough. All the data obtained are available in Genebank, and they are carefully annotated. It discusses the potential role of *Anopheles gibbins*i in malaria transmission in an area where the primary vector is close to extinction. Currently, there are only a few DNA sequences available for studying this species. Providing a complete mitochondrial genome will significantly aid in understanding its relationships with other *Anopheles* species resolving possible cryptic relations, and quickly identifying and monitoring this potential malaria vector. Data collection and sequencing are well-described and reproducible. The data collection and sequencing methods are well-documented and reproducible. The phylogenetic analysis was conducted using only the 13 protein-coding genes and presented an updated understanding of the relationships among *Anopheles gibbinsi and *different species in the *Cellia* clade.

 I have a few suggestions for improving the clarity and delivery of the paper:

 1. Consider enhancing the mitochondrial dataset for phylogenetic reconstruction by including additional mitochondrial sequences from the subgenus *Cellia*. This could provide better insight into the relationship between this species and others within the *Cellia* group, as well as with the primary Plasmodium vector.

 2. It would be helpful to include the distance to the tree. The current tree structure appears to be an ultrametric tree.

Are the datasets clearly presented in a usable and accessible format, and the assembly and annotation available in an appropriate subject-specific repository?

Yes

Are sufficient details of the sequencing and extraction, software used, and materials provided to allow replication by others?

Yes

Are the rationale for sequencing the genome and the species significance clearly described?

Yes

Are the protocols appropriate and is the work technically sound?

Partly

Reviewer Expertise:

My research area is population genomics and molecular phylogenetics. I have worked on Aedes species mitochondrial phylogeny and related flaviviruses.

I confirm that I have read this submission and believe that I have an appropriate level of expertise to confirm that it is of an acceptable scientific standard.
